# Sensitivity Analysis and Impact of the Kappa‐Correction of Residual Ionospheric Biases on Radio Occultation Climatologies

**DOI:** 10.1029/2019EA000942

**Published:** 2020-07-16

**Authors:** J. Danzer, M. Schwaerz, G. Kirchengast, S. B. Healy

**Affiliations:** ^1^ Wegener Center for Climate and Global Change (WEGC) University of Graz Graz Austria; ^2^ Institute of Physics, NAWI Graz University of Graz Graz Austria; ^3^ European Centre for Medium‐range Weather Forecasts (ECMWF) Reading UK

## Abstract

A new model was recently introduced to correct for higher‐order ionospheric residual biases in radio occultation (RO) data. The model depends on the *α*_1_ and *α*_2_ dual‐frequency bending angle difference squared, and a factor *κ*, which varies with time, season, solar activity, and height, needing only the F_10.7_ solar radio flux index as additional background information. To date, this kappa‐correction was analyzed in simulation studies. In this study, we test it on real observed Metop‐A RO data. The goal is to improve the accuracy of monthly mean RO climate records, potentially raising the accuracy of RO data toward higher stratospheric altitudes. We performed a thorough analysis of the kappa‐correction, evaluating its ionospheric sensitivity during the solar cycle for monthly RO climatologies and comparing the kappa‐corrected RO stratospheric climatologies to three other data sets from reanalysis and passive infrared sounding. We find a clear dependence of the kappa‐correction on solar activity, geographic location, and altitude; hence, it reduces systematic errors that vary with the solar cycle. From low to high solar activity conditions, the correction can increase from values of about 0.2 K to more than 2.0 K at altitudes between 40 to 45 km. The correction shifts RO climatologies toward warmer temperatures. With respect to other data sets, however, we found it difficult to draw firm conclusions, because the biases in the other data sets appear to be at similar magnitude as the size of the kappa‐correction. Further validation with more accurate data will be useful.

## Key Points

- The kappa‐correction model is tested for the impact of its correction of higher‐order ionospheric biases in radio occultation data
- The magnitude of the kappa‐correction varies over the solar cycle from 0.2 K to more than 2.0 K at about 40 km
- The correction shifts the stratospheric data toward warmer temperatures; firm validation based on other data sets was difficult

## 1 Introduction

The global navigation satellite system (GNSS) radio occultation (RO) technique (Hajj et al., 2002; Kursinski et al., 1997) has become a valuable source of geophysical data over the past years. A continuous time series exists since the launch of the CHAllenging Minisatellite Payload (CHAMP) satellite mission in the year 2001 (e.g., Ao et al., 2003; Foelsche et al., 2003; Wickert et al., 2001). The data have applications in numerical weather prediction (NWP) (e.g., Cardinali, 2009; Healy & Thépaut, 2006) and also climate research and climate monitoring of the Earth's atmosphere (e.g., Ho et al., 2012; Steiner et al., 2011, 2013).

RO data have high accuracy over the upper troposphere and lower stratosphere between about 5 to 35 km (e.g., Foelsche et al., 2011; Kursinski et al., 1997). Toward higher altitudes, measurement noise and the influence of the ionosphere become an increasing error source in the RO data. The ionospheric influence is usually corrected to first order by a dual‐frequency linear combination of RO bending angles (Ladreiter & Kirchengast, 1996; Vorob'ev & Krasil'nikova, 1994). Nevertheless, residual ionospheric errors (RIE) remain, leading to systematic biases in the data, increasing in relevance at altitudes above about 35 km (Danzer et al., 2013; Liu et al., 2013, 2018, 2015; Mannucci et al., 2011; Syndergaard, 2000). Different approaches for higher‐order ionospheric corrections exist (e.g., Hoque & Jakowski, 2008; Kedar et al., 2003; Syndergaard, 2002; Vergados & Pagiatakis, 2010, 2011), having the disadvantage of the need for additional background information, such as the electron density in the vicinity of the ray path or the geomagnetic field.

Recently, a new model approach for the correction of higher‐order ionospheric residuals was introduced by Healy and Culverwell (2015), the so‐called kappa‐correction. Healy and Culverwell (2015) derived the kappa‐correction from an integral expression for the RIE based on the original work by Vorob'ev and Krasil'nikova (1994). The resulting kappa‐correction consists of a product of two factors: The first factor, *κ*, shows only moderate variations, dependent on altitude, solar activity, local time, and season, while the second factor depends only on the dual‐frequency bending angles *α*_1_ and *α*_2_, capturing the ionospheric variations from profile to profile.

The aim of this work is to test the kappa‐correction for climatological applications. The goal is to improve the accuracy of monthly mean climatologies, potentially raising the current limit in quality of RO data from stratospheric altitudes of about ∼35 km to altitudes of about ∼45 km. To date, the kappa‐correction was tested in two simulation studies (Angling et al., 2018; Danzer et al., 2015) using the NeUoG ionosphere model (Leitinger & Kirchengast, 1997) and the NeQuick model (Nava et al., 2008), respectively. The main advantage of the kappa‐correction is that it is only based on the two measurable quantities, the *α*_1_ and *α*_2_ bending angles, and the F_10.7_ index for the solar activity. Information about the electron density or the geomagnetic field are not needed. For that reason, the kappa‐correction is quick and easy to apply at bending angle level to each RO profile.

We combine the kappa‐correction with the average‐profile‐inversion (API), where profiles are averaged in bending angle space, and the average profiles are then passed into the further processing (Danzer et al., 2014, 2018; Gleisner & Healy, 2013). The API method has the advantage of reducing the influence of background in the data, since the average profiles can be used up to about 80 km. Climatological products of refractivity, density, pressure, and temperature are directly produced in the retrieval process from those average bending angle profiles, instead of obtaining individual RO profiles. In previous studies (Danzer et al., 2015; Healy & Culverwell, 2015), it has been suggested to combine the kappa‐approach with the API method. The reason is that the kappa‐correction does not correct for errors caused by horizontal gradients or by the Earth's geomagnetic field. These errors produce noise for individual profiles, but are assumed to average out in the context of climatologies.

This work is structured in the following way: First, we introduce the method and the data sets (section 2). Afterwards, we compute the kappa‐correction for each individual profile and discuss filtering and smoothing steps for the RIE (section 3.1). As a next step, we perform a thorough sensitivity study of the kappa‐correction through the solar cycle from the solar minimum year 2008 up to the solar maximum year 2015. We analyze the sensitivity of the correction with altitude and latitude for bending angle and temperature RO climatologies (section 3.2). In a final step, we compare RO temperature climatologies with and without the kappa‐correction with three other data sets from the European Center for Medium‐range Weather Forecast Re‐Analysis Interim (ERA‐Interim) (Dee et al., 2011), ERA5 (Hersbach et al., 2018), and the Michelson Interferometer for Passive Atmospheric Sounding (MIPAS) (Fischer et al., 2008; Wang et al., 2004) (section 3.3). We conclude with a discussion of the results in section 4.

## 2 Method and Data Sets

### 2.1 Method

We combine two different approaches in the production of RO climatologies, with the goal to improve data quality in the stratosphere. The two methods will be introduced briefly here, mainly referring to the original work.

The key research question in this study is the impact of the kappa‐correction on RO climatologies. In a first step, we apply the higher‐order ionospheric correction on the individual first‐order ionosphere corrected Metop‐A RO bending angle profiles. The estimate of the neutral atmosphere bending angle (*α*_*c*_) according to Vorob'ev and Krasil'nikova (1994) is given by

(1) $$\alpha_{c}(z_{a})=\alpha_{L_{1}}(z_{a})+\frac{f_{2}^{2}}{f_{1}^{2}-f_{2}^{2}}[\alpha_{L_{1}}(z_{a})-\alpha_{L_{2}}(z_{a})]\;,$$

where $\alpha_{L_{1}}$ and $\alpha_{L_{2}}$ are the *L*_1_ and *L*_2_ bending angles related to the frequencies *f*_1_ and *f*_2_, given at impact altitude *z*_*a*_. Equation 1 is a correction to the first‐order ionospheric effect, neglecting higher‐order ionospheric residuals that depend on the GNSS signal frequency, the Earth's magnetic field, and electron number density concentration along the signal propagation (e.g., Danzer et al., 2013; Liu et al., 2013, 2018, 2015). It produces a small negative bending angle error even for a simple spherically symmetric ionosphere, where the geomagnetic term is neglected. Therefore, Healy and Culverwell (2015) propose a modification to the standard ionospheric correction, with an additional positive term to compensate for this error:

(2) $$\alpha_{c}(z_{a},t)=\alpha_{L_{1}}(z_{a})+\frac{f_{2}^{2}}{f_{1}^{2}-f_{2}^{2}}[\alpha_{L_{1}}(z_{a})-\alpha_{L_{2}}(z_{a})]+|\kappa (z_{a},t)|\cdot {\left[\alpha_{L_{1}}(z_{a})-\alpha_{L_{2}}(z_{a})\right]}^{2}\;.$$

The latter additional term is the kappa‐correction term, consisting of a product of two factors; one expresses the dominant residual ionospheric variation from profile to profile, the other describes the weak altitudinal variation (*κ* factor). Note the introduction of a temporal dependance *t*, due to applying a solar cycle dependent correction. The main advantage of the kappa‐correction is that the dominant factor depends on two measurable quantities, the difference of the *L*_1_ and *L*_2_ bending angles ( $\alpha_{L_{1}}$, $\alpha_{L_{2}}$) squared, which is proportional to the total electron content squared (TEC^2^).

The factor *κ*(*z*_*a*_,*t*) can be approximated by (Angling et al., 2018)

(3) $$\kappa (z_{a},t)=a+b\cdot F_{10.7}(t)+c\cdot \chi (t)+e\cdot z_{a}\;,$$

depending on impact altitude *z*_*a*_, the daily solar activity (expressed by the F_10.7_(*t*) index given in solar flux units sfu, where 1 sfu =10^−22^ Wm^−2^ Hz^−1^), local time, season, and location (comprised by the solar zenith angle *χ*(*t*), given in rad). *a*,*b*,*c*,*e* are scalars found by fitting the model to the data; see Table 1 and Angling et al. (2018). *κ*(*z*_*a*_,*t*) is given in rad^−1^. Note that the factor *κ* is negatively correlated with solar activity; that is, it is low when solar activity is high, and vice versa. In practice, a scalar factor (scal‐*κ*) of *κ*∼14 rad^−1^ is a reasonable approximation; see Danzer et al. (2015).

**Table 1:** Estimated Model Parameters

Parameter	Unity	Estimated value
a	rad^−1^	15.05
b	rad^−1^ sfu^−1^	−1.243×10^−2^
c	rad^−2^	2.372
e	rad^−1^ km^−1^	−5.332×10^−2^

In a further step, we apply the average‐profile‐inversion (API) to the ionosphere corrected bending angles, analyzing monthly 10° zonal‐mean Metop‐A climatologies (Danzer et al., 2014, 2018; Gleisner & Healy, 2013). In the API method, RO climatologies are built on the bending angle level and the mean bending angle profiles are propagated through the RO retrieval. RO data are in general limited in altitude to below about 80 km and show a decreasing signal‐to‐noise ratio with increasing altitude. For this reason, at the bending angle level, background data (e.g., data from climatological models or from meteorological data) are usually introduced through a statistical optimization (SO) step (e.g., Gobiet & Kirchengast, 2004; Ho et al., 2009, 2012). RO profiles are then individually processed through the retrieval. The problem with the standard method is that different implementations of the SO and structural uncertainties increase through the retrieval chain (Ho et al., 2012; Steiner et al., 2013). The advantage of the API method is that it reduces the influence of background data and circumvents the SO step in the processing, reducing the processing bias in RO climatologies.

### 2.2 Data

For the analysis of the ionospheric kappa‐correction, we use example data from the Metop‐A satellite mission (e.g., Loiselet et al., 2000; Montenbruck et al., 2008; Von Engeln et al., 2009). The kappa‐correction was calculated for each RO profile and afterwards applied to the individual bending angles. For the analysis of the functional kappa (func‐*κ*, Equation 3), the F_10.7_ solar flux data were downloaded from Natural Resources Canada (https://www.spaceweather.gc.ca/solarflux/sx-5-en.php, last access: 13 May 2020).

The RO Metop‐A data were studied on a monthly basis and binned into 10° zonal‐mean climatologies, computed from the solar minimum period (March 2008) up to the solar maximum period (January 2015). We computed three different RO climatology runs for the complete period:

- *κ* equals zero: leading to the standard first‐order ionospheric correction;
- scal‐*κ*: kappa is set to $\kappa =\kappa_{c}=14\;{\text{rad}}^{-1}$;
- func‐*κ*: kappa (*κ*_*f*_) is computed using Equation 3.

For the validation of the data, we use the three other data sets ERA‐Interim, ERA5, and MIPAS. All data sets show high quality at the stratospheric altitudes we are interested in (between about 20 to 45 km). The ERA‐Interim reanalysis (Dee et al., 2011) covers the period 1979 to August 2019. It has a horizontal sampling of 79 km (TL255), and 60 vertical levels from the surface up to 10 Pa. This reanalysis uses a four‐dimensional variational approach (4D‐Var), based on the ECMWF NWP system implemented operationally in 2006 (Cycle 31R2). ERA‐Interim generally assimilate operational satellite data, rather than reprocessed data sets so, for example, the change in the operational COSMIC GNSS‐RO processing in November 2009 led to a small discontinuity in the stratospheric temperatures. The ERA5 system (Hersbach et al., 2018) is based on the NWP system implemented operationally at ECMWF in 2016 (Cycle 41R2). It has better spatial resolution, with a horizontal sampling of 31 km (TL639) and 137 vertical levels from the surface to 1 Pa. ERA5 also makes greater use of reprocessed data sets, including reprocessed Constellation Observing System for Meteorology, Ionosphere, and Climate (COSMIC) GNSS‐RO data. However, both ERA‐Interim and ERA5 assimilate the operational Metop‐A GRAS data provided by EUMETSAT from May 2008. The MIPAS temperature information is not assimilated in either the ERA5 or ERA‐Interim reanalysis.

The consistency of global atmospheric temperature reanalyses—including ERA5 and ERA‐Interim—in the lower and middle stratosphere has improved since around 2006 with the assimilation of GNSS‐RO (Ho et al., 2019; Long et al., 2017; Luntama et al., 2008), but ERA‐Interim is still around ∼1.5 K warmer than ERA5 globally at 1 hPa. The ERA‐Interim and ERA5 data used here are monthly means of daily averages (reanalysis stream = “MODA”), computed at fixed pressure levels.

MIPAS data are available from July 2002 to April 2012 (García‐Comas et al., 2012), so comparisons to Metop‐A data in the later high solar activity months are not possible. A validation study of MIPAS data relative to WEGC RO OPSv5.6 data has been performed on a profile to profile basis by Innerkofler (2015). The systematic temperature difference is found to be within ±1 K up to about 40 km.

## 3 Results

**Figure 1:**
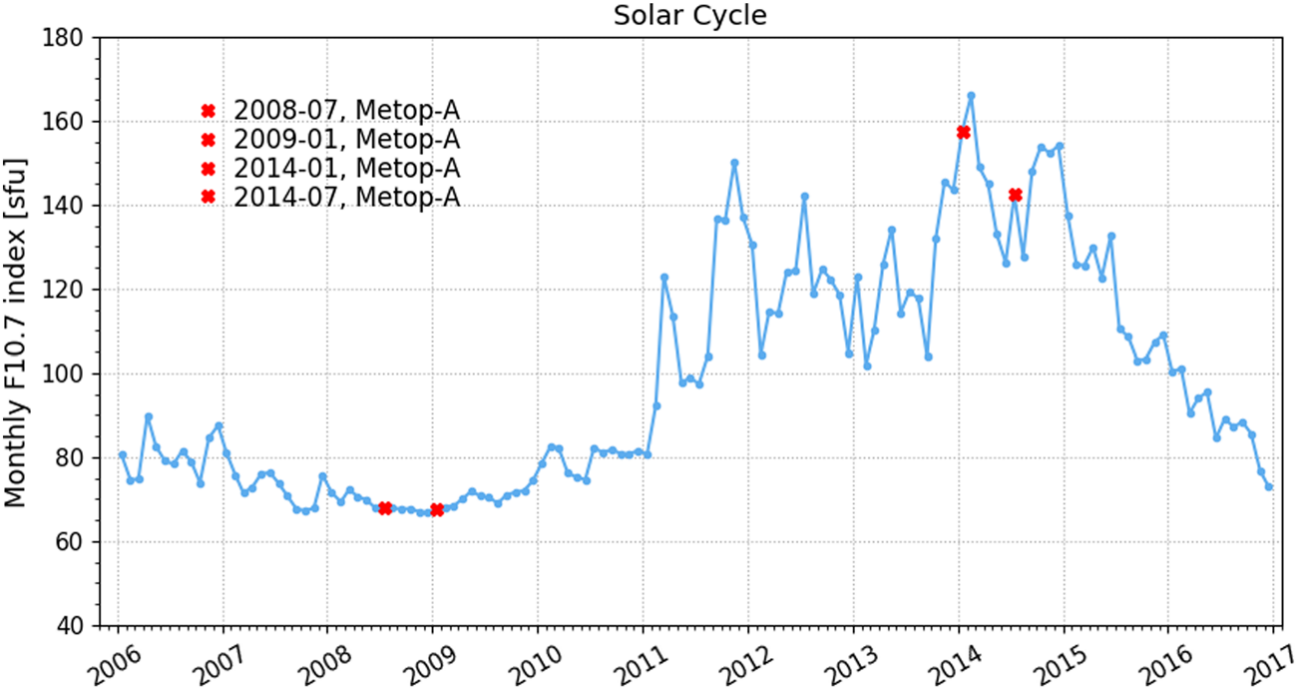
Monthly mean F_10.7_ solar flux values during the solar cycle period 2006 to 2016 and four respective test months (see legend), covering low and high solar activity conditions.

Figure 1 shows solar cycle variations, expressed by the F_10.7_ index as a function of time. In our analysis, we focus on the period from the minimum year 2008 up to the maximum year 2014. This time period catches the minimum to maximum dependence of RO data on solar activity. We also mark in Figure 1 four test months which catch summer and winter—low and high solar activity conditions, that is, July 2008, January 2009, January 2014, and July 2014. In later analysis, we will sometimes directly compare those test months for illustrating the two solar activity extremes in two opposite seasons.

### 3.1 Characteristics of the Kappa‐Correction

**Figure 2:**
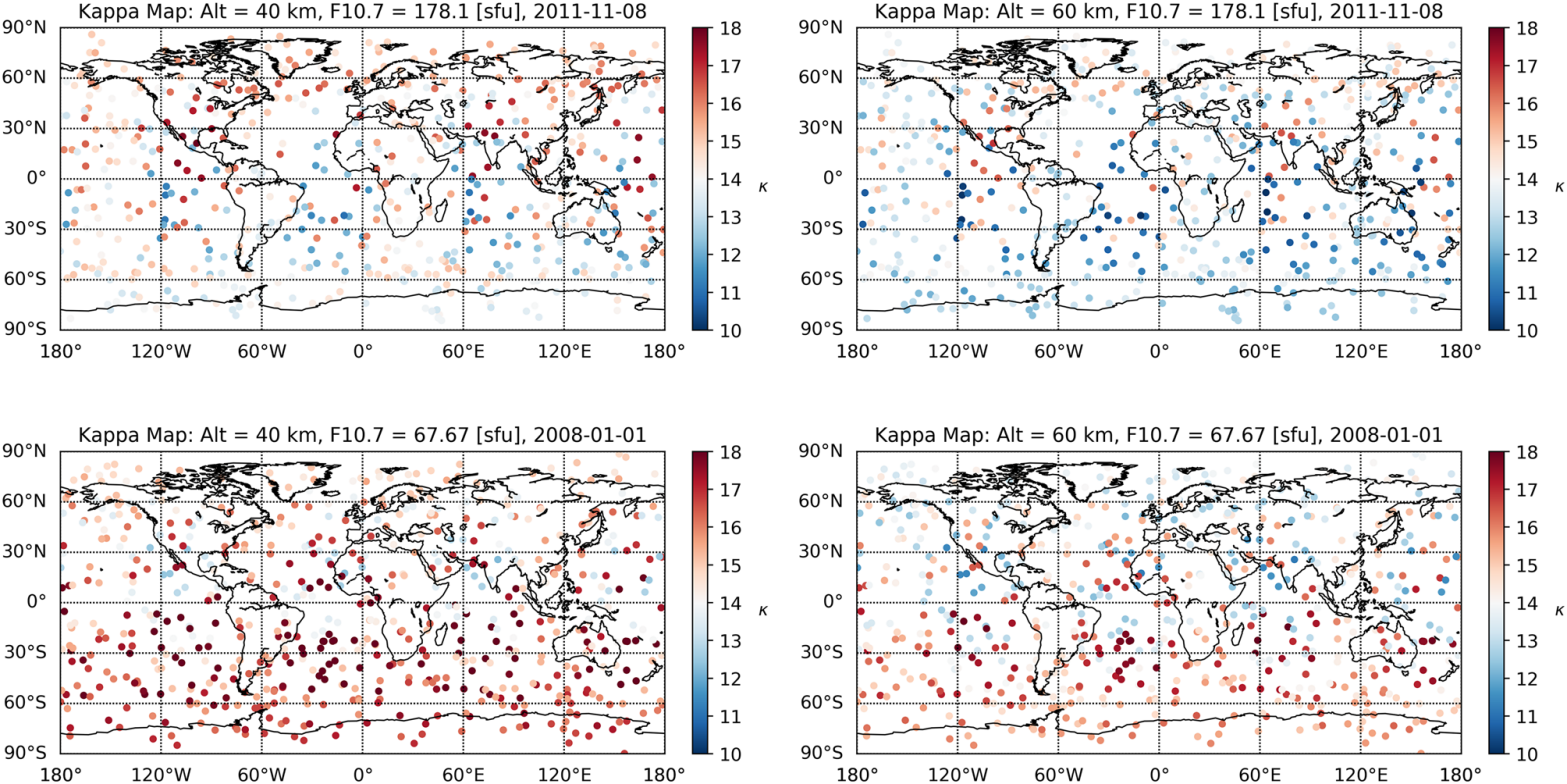
Maps of kappa‐values of a high and a low solar activity day (see panel titles), computed with Equation 3 for Metop‐A RO events at 40‐km (left column) and 60‐km (right column) altitude.

**Figure 3:**
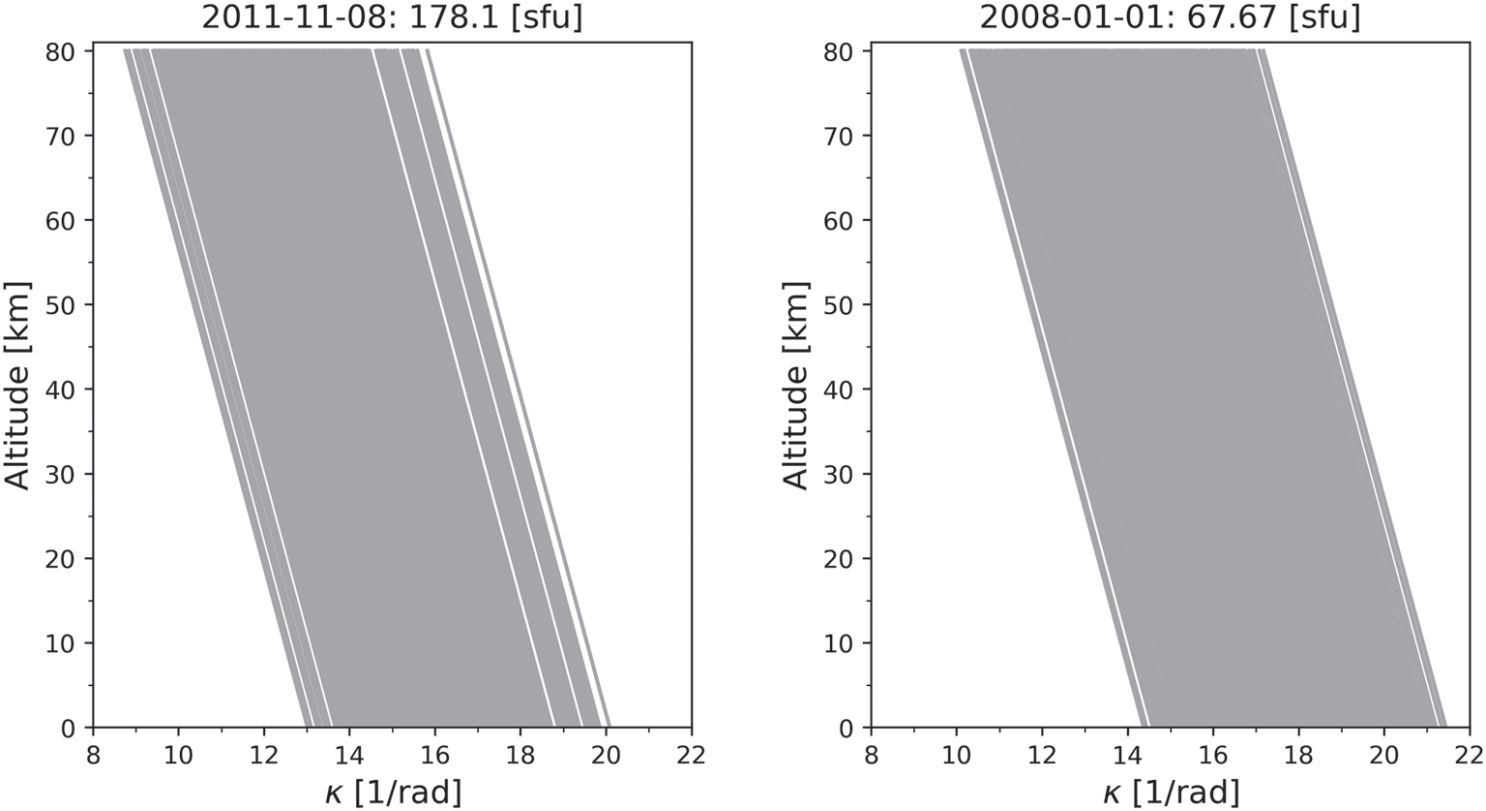
Altitude variation of kappa‐values for a high and low solar activity day (see panel titles), computed with Equation 3.

In an initial analysis, we discuss the computation and preparation of the kappa‐correction. Previous studies computed the kappa‐correction using simulated data (Angling et al., 2018; Danzer et al., 2015), but now we focus on real observed RO data. In Figure 2, we plot a map of kappa values for a high and a low solar activity test day. The computed kappa values clearly decrease with increasing altitude, as well as with increasing solar activity. The linearly decreasing smooth behavior of the kappa values with increasing altitude and F_10.7_ index is also illustrated in Figure 3, indicating the negative correlation of *κ* with solar activity. The kappa values have been computed using Equation 3; hence, the values depend on F_10.7_ index, solar zenith angle *χ*, and (impact) altitude *z*_*a*_.

**Figure 4:**
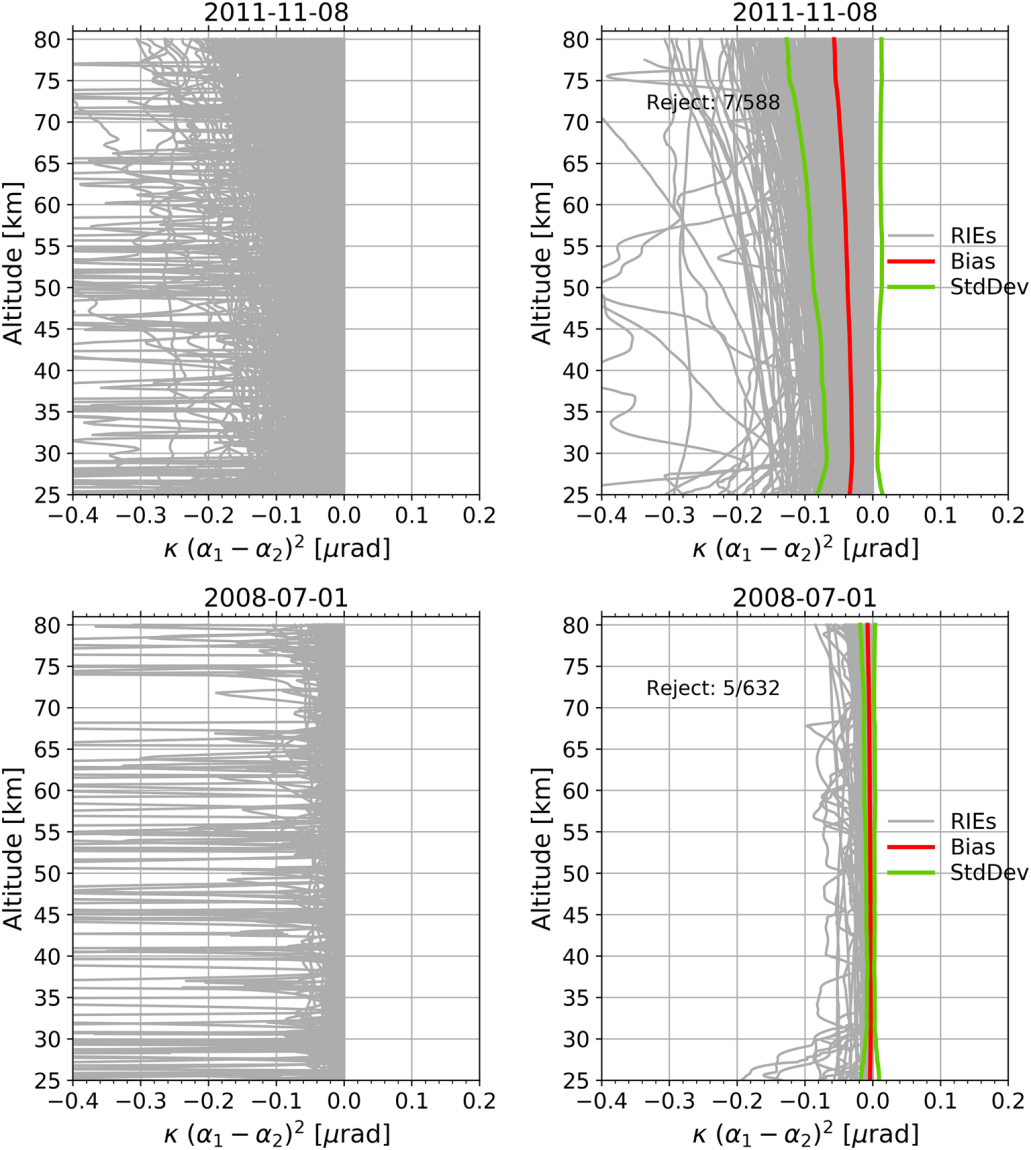
Illustration of the kappa‐correction term, for a high (top row) and low (bottom row) solar activity example day, at different calculation steps: before filtering/smoothing steps (left column), after filtering/smoothing steps (right column).

In a next step, we calculate the complete kappa‐correction term using Equation 2. The kappa‐correction is correlated with solar activity, increasing with the rising solar cycle, as shown in Figure 4, where we compare a solar high (top row) and solar low (bottom row) example day. The RIE shows large fluctuations, introduced through the $\alpha_{L_{1}}$, $\alpha_{L_{2}}$ bending angle difference squared; see left column of Figure 4. For that reason, an outlier correction combined with a moving average filter was applied. Outliers larger than 0.1 μrad^2^ on the $\alpha_{L_{1}}$, $\alpha_{L_{2}}$ difference squared were rejected. Regarding the moving average, a 10‐km window on the $\left(\alpha_{L_{1}}(h)-\alpha_{L_{2}}(h)\right)$ difference was applied. Furthermore, the kappa‐correction was set to zero below the altitude of 30 km, since from this level and below the neutral bending angle magnitude is greater or equal to about 300 μrad and therefore the impact of the kappa‐correction is small. The ionospheric impact on RO data, however, is large at high altitudes and strongly decreases toward the surface. At lower altitudes, the $\alpha_{L_{1}}$, $\alpha_{L_{2}}$ differences show large noise due to neutral atmospheric small‐scale dynamical processes, such as from atmospheric wave activity, in particular the wide spectrum of acoustic‐gravity waves. Altogether we tested three different cut off heights; 25, 30, and 35 km, resulting in negligible differences on the RO climatologies.

Figure 4 shows, for a high and low solar activity test day, the results before and after the filtering and smoothing steps. The outlier correction rejects only a few profiles (7/588‐high solar conditions, 5/632‐low solar conditions) but is still strong enough to remove the largest fluctuations without a major loss of profiles. After the filtering and smoothing step, we find for the low solar activity day already a rather smooth RIE correction for each profile. For the high solar activity day, still some profiles show larger fluctuations. On the one hand, this simply reflects the nature of the more challenging high solar condition days. On the other hand, it does not introduce a problem when the correction is applied to the bending angle profiles. The bending angles are anyhow binned and averaged for the later average‐profile‐inversion, which strongly mitigates the impact of the few profiles with larger fluctuations.

### 3.2 Sensitivity Analysis of the Kappa‐Correction

**Figure 5:**
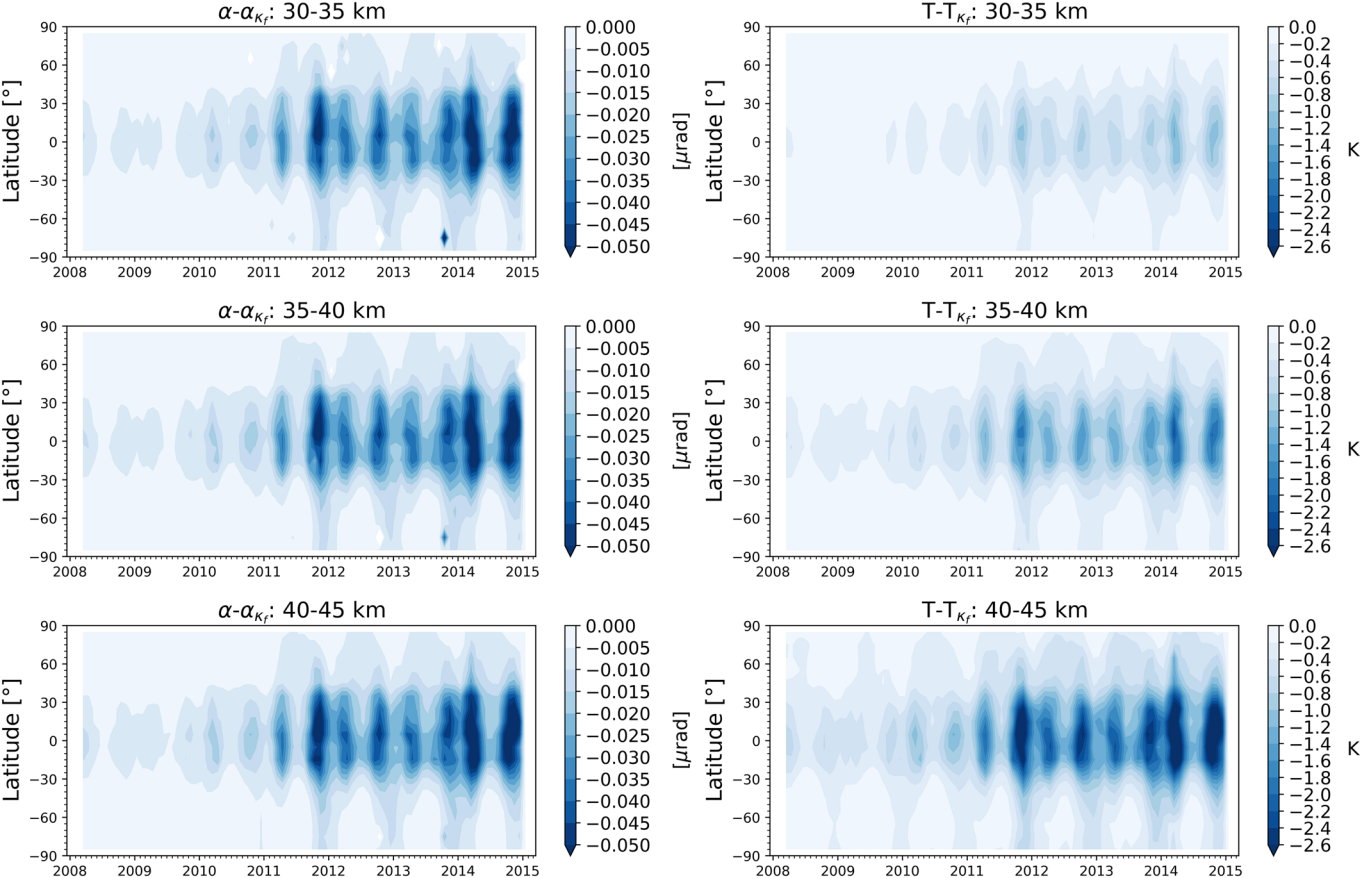
Size of the kappa‐correction term at bending angle retrieval level ( $\alpha -\alpha_{\kappa_{f}}$, left column), and at temperature retrieval level ( $T-T_{\kappa_{f}}$, right column), within three different stratospheric altitude layers (rows; see panel titles).

**Figure 6:**
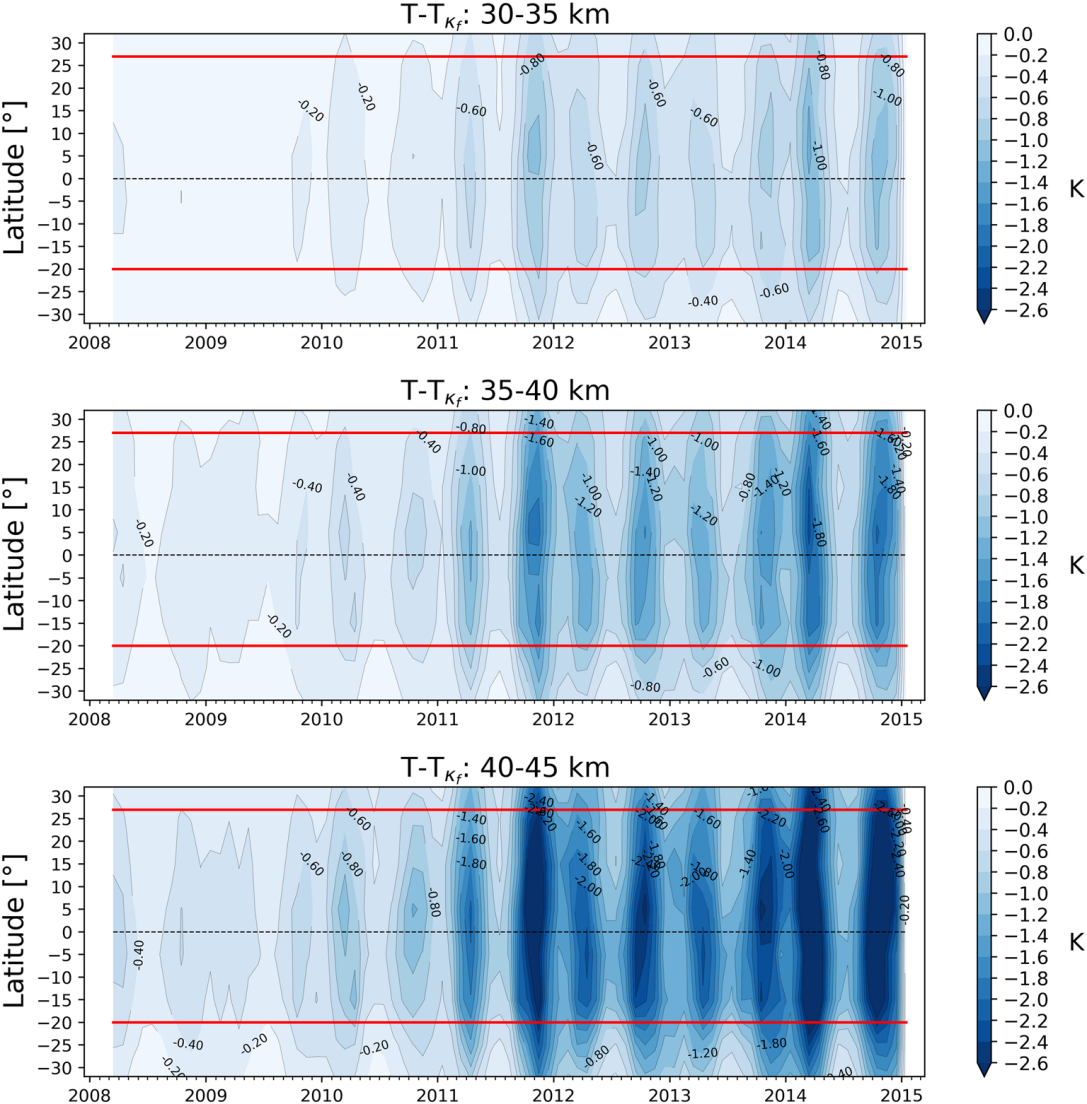
Impact of the kappa‐correction on temperature level as a time series over the tropics. The two red lines indicate the small asymmetry of the kappa‐correction between the northern and southern hemisphere, with respect to the equator (black dashed line).

In this section, we illustrate the sensitivity of the kappa‐correction to the solar cycle. Figure 5 shows the impact of the correction at the bending angle and temperature levels. The kappa‐correction corrects at bending angle level only for very small values at the order of about 10^−8^ rad. Clearly, the values increase in the high solar activity period, and the values are also largest around the tropics. Of specific interest is the resulting impact at temperature level. First, we detect a distinct increase of the impact of the correction toward higher altitudes. Second, the impact of the correction increases with rising solar activity. And third, maximum values of the correction are found around the tropics, where we present a more detailed illustration in Figure 6. At the 30‐ to 35‐km altitude layer, the impact is for the minimum years around −0.2 K, increasing for the rising cycle and the tropics up to −0.6 to −1.0 K. For the 40‐ to 45‐km altitude layer, the difference can be around −2.0 K in the tropics at solar maximum conditions. The impact of the correction shows a slight but not strong shift toward the northern hemisphere. While on the northern hemisphere, the peak of the correction extends until between about 25°N to 30°N (solid red line), the peak on the southern hemisphere extends usually until 20°S (solid red line). In general, the magnitude of the kappa‐correction through the solar cycle is in line with results from our previous simulation study (Danzer et al., 2015). In the simulation study, temperature errors at the tropics increased from −0.2 K up to more than −1.0 K at 35 km. Also, the study of Vergados and Pagiatakis (2011) analyzes the latitudinal, vertical, and solar variation of the second‐order residual ionospheric error of RO atmospheric parameters, however, for 40 individual CHAMP profiles. Given the differences in the approach and sample size, these results appear to be reasonably consistent.

**Figure 7:**
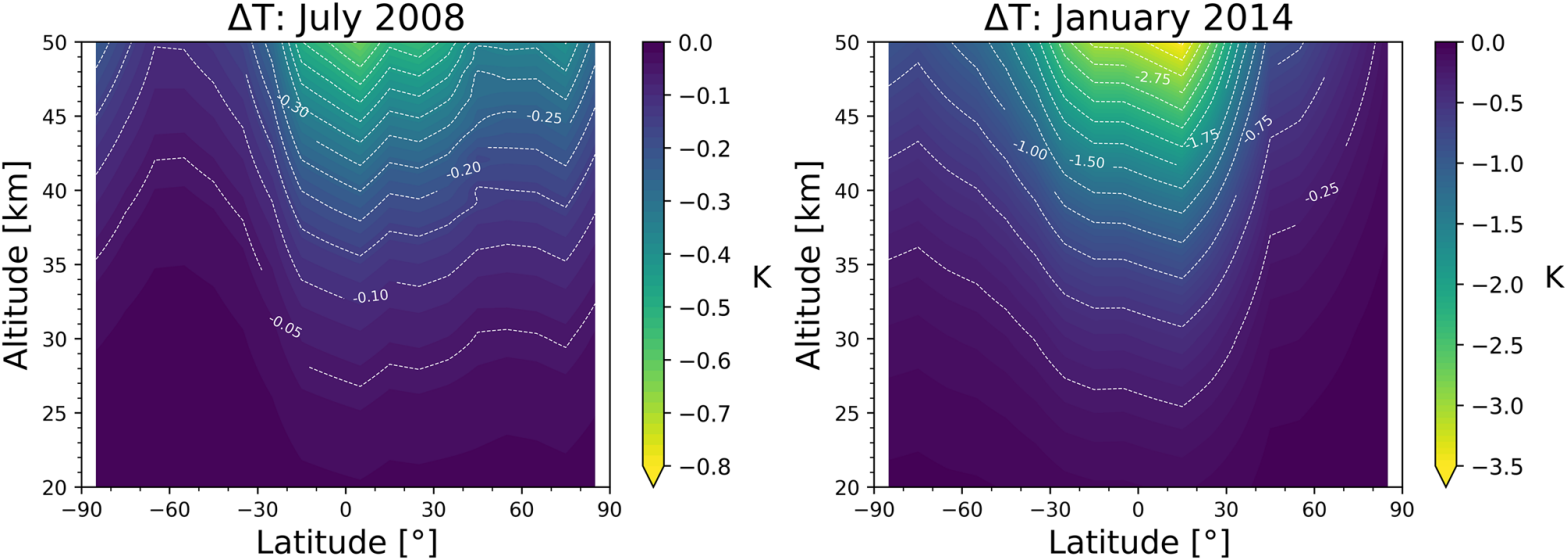
Temperature difference, Δ*T* = *T* − *T*_*κ*_, for a low (left) and high (right) activity test month (see panel titles).

In Figure 7, we compare the temperature difference (Δ*T* = *T* − *T*_*κ*_) between a low and a high solar activity month. While for the low solar activity month, the impact of the correction is less than −0.6 K below 50‐km altitude, for the high activity month, it increases to about −3.5 K at the same altitude level. In Figures 5, 6, 7, one can observe the typical northern hemisphere summer and winter conditions, with the peak of the correction for both seasons in the northern hemisphere equatorial region. This is expected due to the combination of, on the one hand, the shift between the geographic and geomagnetic equator and, on the other hand, the equatorial ionization anomaly, leading to higher ionization levels toward the northern equatorial region (e.g., Balan et al., 2018).

**Figure 8:**
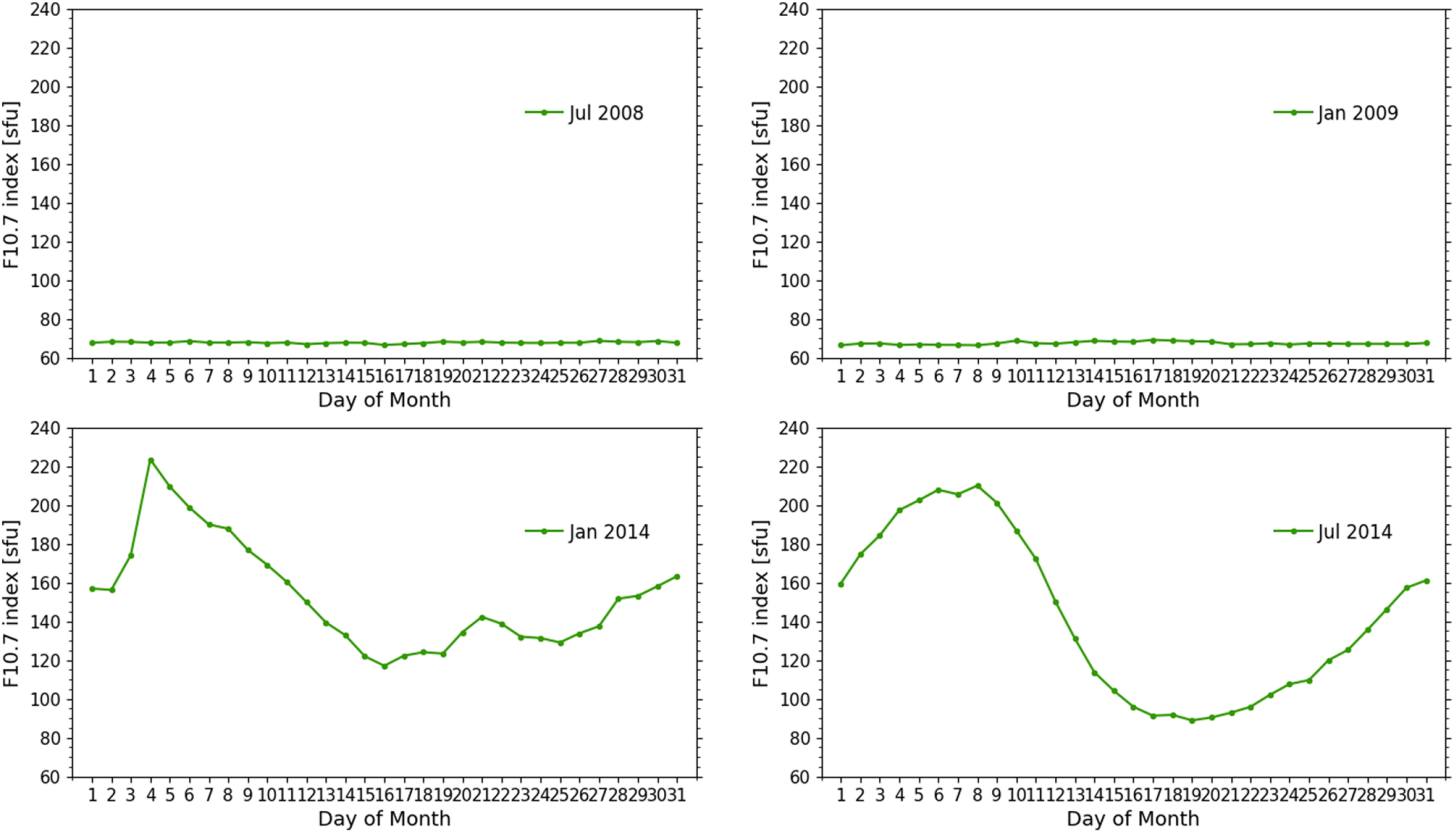
Daily F_10.7_ solar flux values during the four test months, indicated in Figure 1.

**Figure 9:**
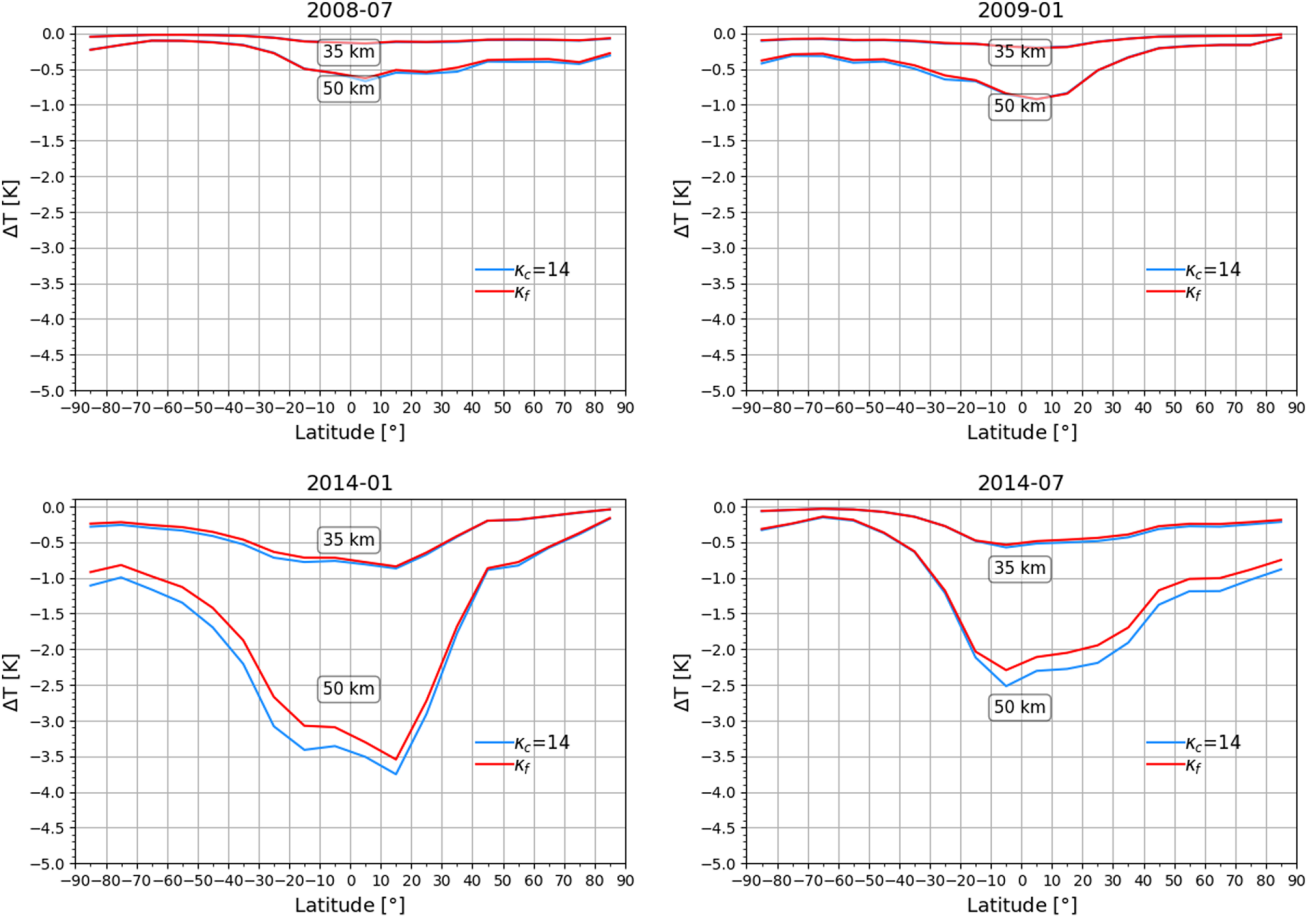
Comparison of the “scal‐*κ*” and “func‐*κ*” results under different ionospheric conditions (four test months; see panel titles), showing zonal‐mean temperature climatology differences for 35‐ and 50‐km altitude levels.

Finally, we discuss scal‐*κ*. We investigate the four months July 2008, January 2009, January 2014, and July 2014, catching winter and summer low and high solar activity conditions. Figure 8 shows the daily F_10.7_ values of the four respective months. The two low solar activity months show constantly low F_10.7_ values, around *F*_10.7_ = 65 sfu. The two high activity months peak around *F*_10.7_ = 220 sfu and show on average an activity of about *F*_10.7_ = 160 sfu. We analyze the difference between RO temperature climatologies computed without kappa‐correction, *T*, and with kappa‐correction, *T*_*κ*_, that is, *T*−*T*_*κ*_ = Δ*T*. Furthermore, we compute the correction using Equation 2; once for scal‐*κ* with $\kappa_{c}=14\;{\text{rad}}^{-1}$, and also for func‐*κ* (*κ*_*f*_) computed according to Equation 3. We show the results in Figure 9. For the low solar activity months, we find $T-T_{\kappa_{c}}$ and $T-T_{\kappa_{f}}$ almost identical for altitudes of 35 and 50 km. The impact of the correction at 35 km is small (approximately −0.2K), and at 50 km, it increases from −0.6 to −0.9 K (top row).

For the high solar activity months, the difference between the *κ*_*c*_ and *κ*_*f*_ corrections during hemispherical summer and within the tropics (±20°) are distinct, and these differences are more pronounced at 50‐km altitude. During hemispherical winter, the two kappa‐correction techniques present the same results at both altitudes studied here. The analysis of Angling et al. (2018) suggests that a functional modeling of kappa reproduces the kappa found in 25,000 simulations more accurately. It seems that a scalar kappa produces a larger second‐order ionospheric correction toward higher altitudes and for northern and southern hemisphere summer season.

### 3.3 Comparison to Other Data Sets

In this section, we compare the ionospheric‐corrected RO temperature climatologies against modern reanalyses and observation. We use ERA‐Interim, ERA5, and MIPAS monthly 10° zonal‐mean climatologies. In general, it was difficult to find comparison data sets which show very high quality at stratospheric altitudes between about 25 to 40 km. We want the accuracy of the comparison data sets to be such that we can assess whether the kappa‐correction improves or degrades the GNSS‐RO climatologies. The three chosen data sets have reasonably high quality stratospheric data.

**Figure 10:**
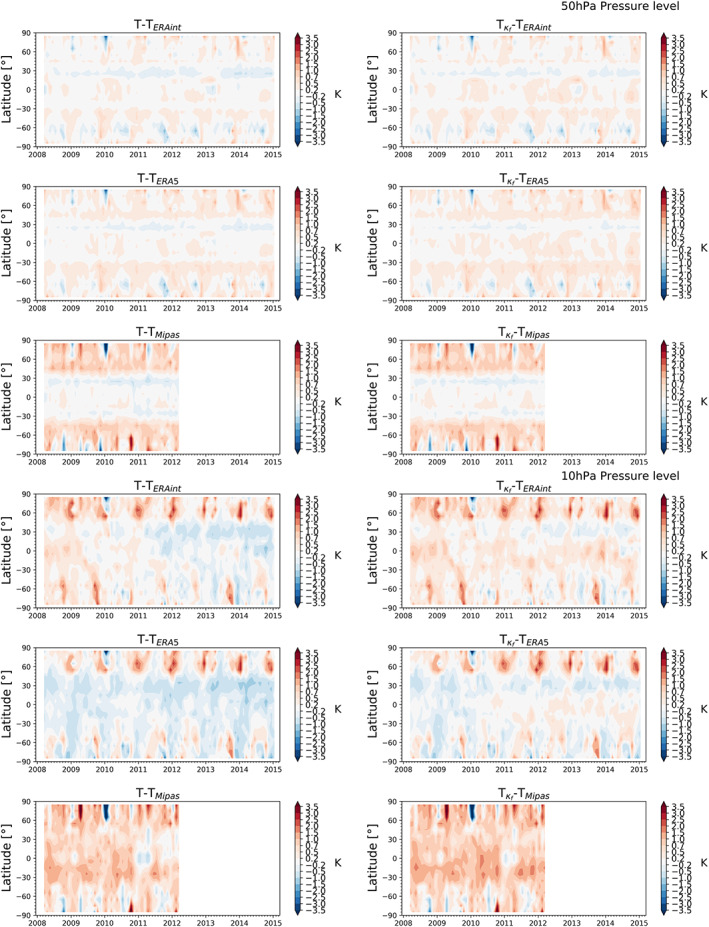
Temperature difference of lower stratosphere zonal‐mean RO temperature climatologies within March 2008 to January 2015 relative to ERA‐Interim (top), ERA5 (middle), and MIPAS (bottom), shown at the 50‐hPa pressure level (top panels, ∼21‐km altitude) and 10‐hPa pressure level (bottom panels, ∼31‐km altitude).

**Figure 11:**
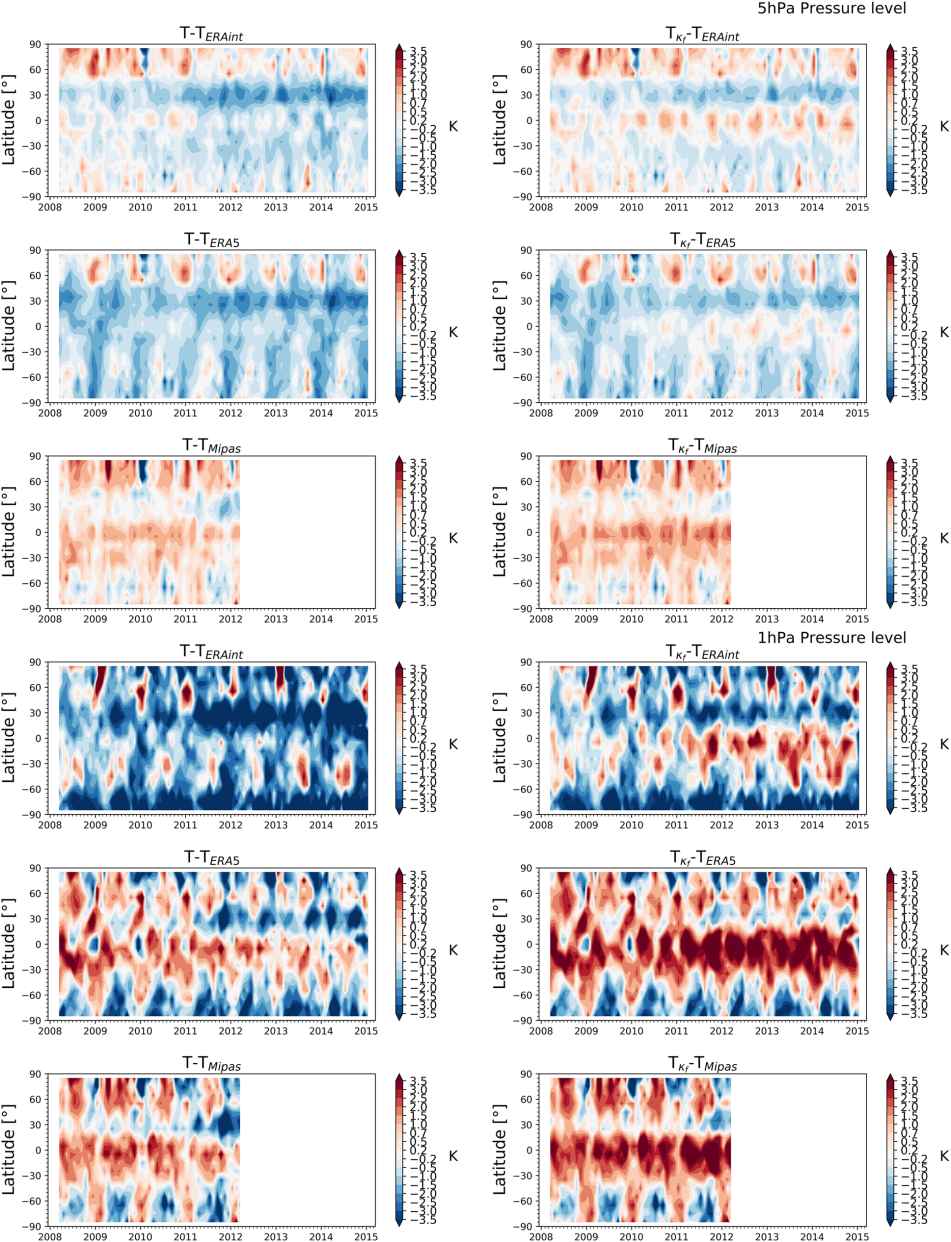
Temperature difference of upper stratosphere zonal‐mean RO temperature climatologies within March 2008 to January 2015 relative to ERA‐Interim (top), ERA5 (middle), and MIPAS (bottom), shown at the 5‐hPa pressure level (top panels, ∼35‐km altitude) and 1‐hPa pressure level (bottom panels, ∼48‐km altitude).

Figures 10 and 11 show the temperature differences between RO temperature climatologies without kappa‐correction (left column), and including the kappa‐correction (right column). The results are given at four pressure levels, 50, 10, 5, and 1 hPa, relative to ERA‐Interim, ERA5, and MIPAS.

Temperature differences relative to other data in the lower stratosphere at the 50‐hPa pressure level are within ±0.2 K for the reanalyses (top panels, Figure 10). The MIPAS differences increase from the midlatitudes toward the poles slightly positive to about 0.5 to 0.7 K (left column, top panels). The larger differences in the northern winter in January 2010 reflected in all three data sets are due to a sudden stratospheric warming (SSW) (e.g., Dörnbrack et al., 2012). In general, the different high‐altitude initializations of RO data have the tendency to somewhat smooth strong anomalies from about 35 km upwards (Angerer et al., 2017; Schwarz et al., 2017); that is, their magnitude can be underestimated for features such as strong SSWs. On the other hand, the reanalysis and MIPAS data sets have their own biases (García‐Comas et al., 2012; Simmons et al., 2019) and may slightly overestimate anomalies. At this pressure level, the kappa‐correction does not have a large impact on the climatologies (right column, top panels). However, we still can see that negative differences slightly decrease when applying the kappa‐correction; see, for example, time series at the latitude band 30°N.

At the 10‐hPa pressure level (bottom panels, Figure 10), the differences increase with respect to other data sets. Negative differences can amount up to −0.5 K, positive differences relative to MIPAS data partly increase to between about 0.7 to 1.0 K. The impact of the correction on the climatologies becomes more evident (right column). Negative differences clearly decrease, but positive differences increase (see differences relative to MIPAS). This indicates that RO temperature data move toward warmer temperatures when the kappa‐correction is applied. Interestingly, in the high solar activity years (starting around the year 2011), the sign changes in the differences relative to ERA‐Interim (from positive to negative, left column). For ERA5, temperature differences are always negative, but slightly increase in the high solar activity years. After applying the kappa‐correction, temperature differences are dominantly in the range of ±0.2 to ±0.5 K (bottom panels, right column).

The general pattern stays the same in the upper stratosphere. At the 5‐hPa pressure level (top panels, Figure 11), negative differences increase relative to ERA‐Interim and ERA5 to about −1 to −1.5 K. In addition, temperature biases slightly increase with rising solar cycle. In general, agreement is again better when including the higher‐order ionospheric correction, moving to between about −0.5 to −1.0 K (right column). Relative to MIPAS agreement is also quite good, however; the bias shows in general a positive tendency, while ERA‐Interim and ERA5 biases tend to be negative. The northern midlatitudes show, to MIPAS data, good agreement around 0.2 K. Between about 10°N to about 50°S, the bias is around 0.2 to 1.0 K.

Finally, we study the 1‐hPa pressure level (bottom panels, Figure 11). The temperature differences are now strongly pronounced, showing also varying differences during the rising solar cycle. RO temperature data including the kappa‐correction show again the shift toward warmer temperatures (bottom panels, right column). In the comparison with ERA‐Interim, this has the consequence that obviously negative differences decrease (bottom panels, top row). However, in the comparisons to ERA5 and MIPAS, the sign of the biases flips, and positive biases become more enhanced after including the kappa‐correction. The biases increase even more strongly in the high solar activity years.

Overall, the largest impact of the kappa‐correction was found over the tropical belt and in geographical regions near 30°N, as expected from Figures 5 and 6. This is due to the equatorial ionization anomaly (EIA) which leads to larger ionization “bulks” northward and southward of the equator (Balan et al., 2018). In general, the EIA strongly depends on local time, having an ionization maximum in the afternoon and evening. Nevertheless, the EIA is also clearly seen in the analysis of climatologies. The impact of the EIA on the residual ionospheric error for individual RO profiles was also investigated by Liu et al. (2013, 2015), showing also the strong increase in the residual bias. With respect to the intercomparison analysis, the kappa‐correction led to a warming of the RO data, resulting in a general decrease of negative differences and an increase of positive differences relative to other data sets.

## 4 Conclusions

RO data have high quality from the upper troposphere to the middle stratosphere (e.g., Steiner et al., 2019). Toward higher altitudes, the impact of errors resulting from measurement noise and ionospheric residuals increases. Usually, ionospheric errors are corrected to first order at bending angle level, leaving residual ionospheric errors of about 6–7*%* of the neutral value at 60 km for solar maximum conditions (e.g., Healy & Culverwell, 2015). Noise and higher‐order ionospheric effects are then reduced at the processing centers through different high‐altitude initializations, using different background data. This study aimed to improve the quality of stratospheric climatologies. To achieve this, we combined the average‐profile‐inversion technique with the ionospheric kappa‐correction. The study provided for the first time a thorough sensitivity analysis for the kappa‐correction through the solar cycle from the solar minimum year 2008 up to the solar maximum until 2015, using Metop‐A RO data.

Results show that the kappa‐correction warms the climatology by about 0.2 up to 1.0 K in an middle stratosphere altitude range from 30 to 35 km, with the largest warming over the tropical belt. In the upper stratosphere altitude range from 40 to 45 km, the ionospheric correction implies to a warming of about 2.0 K under solar maximum conditions.

Interesting in that respect is how RO data compare relative to other data sets. We used three different data sets for our analysis: ERA‐Interim, ERA5, and MIPAS. One of the key challenges was to find data sets with errors sufficiently small in the stratosphere, so that we can assess the impact of the kappa‐correction in the evaluation. Summarizing the intercomparison results, we found the following behavior: First, temperature biases relative to other data sets increase with increasing altitude. Second, temperature biases relative to other data sets vary also with the rising solar cycle. Third, ERA‐Interim and ERA5 temperature data tend to be warmer than RO temperature data (*T*_RO_−*T*_ERA_<0). Fourth, MIPAS temperature data tend to be colder than RO temperature data (*T*_RO_−*T*_MIPAS_>0). Finally, after applying the kappa‐correction to RO data, ERA‐Interim and ERA5 in general tend to agree better, while agreement to MIPAS data tends to decrease.

These mixed results illustrate how challenging it can be to validate potential improvements of RO data quality in the stratosphere. This is likely to be the case for other proposed changes to GNSS‐RO processing in the future. Other data sets have their own biases in the stratosphere, and higher‐order ionospheric corrections are relatively small. In this context, it will be interesting to compare different approaches which correct for higher‐order ionospheric residual biases. More sophisticated approaches, which need additional background information, such as, for example, information about the geomagnetic field, are especially interesting in a profile‐to‐profile comparison study (Liu et al., 2019). In a climatological context, we expect that differences mostly average out between more advanced approaches and the rather simple kappa‐correction; at least in global or zonal‐mean large‐scale averages. The impact of the geomagnetic term as well as the inbound and outbound electron density asymmetries along the RO ray paths will be analyzed in follow‐up investigations.

Finally, we conclude that the ionospheric kappa‐correction is a very simple and operationally fast applicable approach to correct RO profiles on bending level for ionospheric residuals. The main ionospheric variation is captured by using only RO observational data itself and the daily F_10.7_ index, leading to a solar activity‐, local time‐, geographic location‐, season‐, and altitude‐dependent correction. We emphasize the importance of the kappa‐correction method in operational applications, postprocessing climatological analyses, and the potential extension of the radio occultation profiles to higher altitudes above the middle stratosphere. We hope that the improvements reported in this study will aid the quality of future reprocessings of stratospheric climate data records from RO, for the benefit of atmospheric and climate science.

## Data Availability

All RO climatology runs are available at the website (https://wegcowncloud.uni-graz.at/s/ATSqBf4wWqU9T18). The ERA5 and ERA‐Interim reference data can be downloaded online (https://cds.climate.copernicus.eu/#!/search?text=ERA5&type=dataset and https://apps.ecmwf.int/datasets/, respectively). MIPAS data is available online (https://earth.esa.int/web/guest/data-access/view-data-product/-/article/mipas-atmospheric-pressure-temperature-data-constituents-profiles-1547). The F_10.7_ solar flux values were downloaded from Natural Resources Canada (https://www.spaceweather.gc.ca/solarflux/sx-5-en.php, last access: 13 May 2020).
